# The identification of switch-like alternative splicing exons among multiple samples with RNA-Seq data

**DOI:** 10.1371/journal.pone.0178320

**Published:** 2017-05-25

**Authors:** Zhiyi Qin, Xuegong Zhang

**Affiliations:** 1 MOE Key Laboratory of Bioinformatics, Bioinformatics Division and Center for Synthetic and Systems Biology, TNLIST / Department of Automation, Tsinghua University, Beijing, China; 2 School of Life Sciences, Tsinghua University, Beijing, China; Southwest University, CHINA

## Abstract

Alternative splicing is an ubiquitous phenomenon in most human genes and has important functions. The switch-like exon is the type of exon that has a high level of usage in some tissues, but has a low level of usage in the other tissues. They usually undergo strong tissue-specific regulations. There is still a lack a systematic method to identify switch-like exons from multiple RNA-seq samples. We proposed a novel method called iterative Tertile Absolute Deviation around the mode (iTAD) to profile the distribution of exon relative usages among multiple samples and to identify switch-like exons and other types of exons using a robust statistic estimator. We validated the method with simulation data, and applied it on RNA-seq data of 16 human body tissues and detected 3,100 switch-like exons. We found that switch-like exons tend to be more associated with Alu elements in their flanking intron regions than other types of exons.

## Introduction

Alternative splicing (AS) is a major mechanism for increasing the functional complexity and diversity of proteins made from the relatively small number of genes in high eukaryotes especially in human [1, 2]. Different compositions of exons produce multiple transcript isoforms from the same gene by alternative splicing events like exon-skipping, alternative 5’-splice site, alternative 3’-splice site and intron-retention [2]. Isoforms of the same gene are not expressed equally in different cell types and many of them are expressed in tissue-specific manners, which causes different relative usages of alternative exons across different samples. The relative usage of an alternative splicing exon is usually described by its inclusion level, defined as the proportion of expressed transcripts that includes the exon among all expressed transcripts of the gene in the sample [2, 3]. Xing and Lee studied the special type of “tissue-switched” exons that have dramatic changes in inclusion levels across different tissues based on microarray data [3]. Those exons tend to be always included in the expression (i.e., with very high inclusion levels) in some tissues but are always excluded in some other tissues (with 0 or very small inclusion levels), which indicts strong tissue-specific regulations. In the study of human transcriptomes using RNA-sequencing (RNA-Seq) technology, Wang et al observed that genes containing this type of “switch-like” exons “are likely to contribute to fundamental differences in the biology of different human tissues” [2]. A particular feature for detecting “switch-like” exons was included in the MATS method and was designed for comparing two groups of samples [4].

This kind of “switches” play important regulatory roles in many biological processes [5]. For example, Kalsotra et al found that switch-like exons can drive cellular differentiation in heart development [6]. In [7], Venables et al identified 15 switch-like exons between the fibroblastic and pluripotent states related with the differentiation and reprogramming of stem cells [7]. Some individual switch-like exons have been reported to play important roles in health and disease. For example, splicing of Ron exon 11 was found to be altered in breast and colon tumors [8]. Warzecha et al reported that many ESRP-regulated alternative splicing exons switch in their relative usages during epithelial–mesenchymal transition [9], which is implied in tumor metastasis [10].

Not all alternative splicing exons are used in such switch-like manner across samples. It has been observed that switch-like exons are more likely to be ancient exons that has existed for long in evolution, and also more likely to be frame-preserving alternative exons that do not change the reading frame of downstream exons [2, 3]. With the rapid development of next-generation sequencing or NGS technology, more and more RNA-Seq data are being generated for the quantitative study of alternative splicing in many biological problems [2, 11–13]. These RNA-Seq data provide a new opportunity for studying switch-like splicing events [2, 14].

Existing methods used to identify switch-like exons in the above studies were based on criteria decided heuristically by the authors [2, 3]. For example, an exon is identified as a switch-like exon if it is found to have a low inclusion level in some samples but always have a high inclusion level in other samples. This criterion is sensitive to accidental changes in inclusion levels that may be due to noises in the sequencing or mapping steps. A better-defined criterion was introduced in MATS that uses a statistic to identify switch-like exons [4]. But it was only designed for the comparison of two groups of samples [4]. Well-defined methods for detecting switch-like exons among multiple samples based a systematic profiling of relative usage patterns of alternative splicing exons are still lacking.

In this work, we summarized the major patterns of exon relative usage measured by comparing their inclusion levels in multiple groups of samples and proposed a novel method to identify switch-like exons among multiple samples. We call it iTAD or iterative Tertile Absolute Deviation around the mode. It used a robust statistic estimator to analyze the frequency profile of exon relative usage across multiple samples, and to reveal the intrinsic characteristic of switch-like usage patterns comparing to other usage patterns. Therefore it can identify switch-like exons in a more objective manner and can avoid the use of arbitrarily set cutoffs. We conducted a series of simulation experiments to show the accuracy of our method and its robustness to noises. We then applied the method on the RNA-seq data of 16 human tissues, and identified 3,100 significant “switch-like” exons. We also observed that there is not a significant trend for switch-like exons to be formed by Alu elements, but switch-like exons are more likely to be associated with Alu elements in their flanking intron regions than other types of exons.

## Methods

### Overview of iTAD

**Fig 1:**
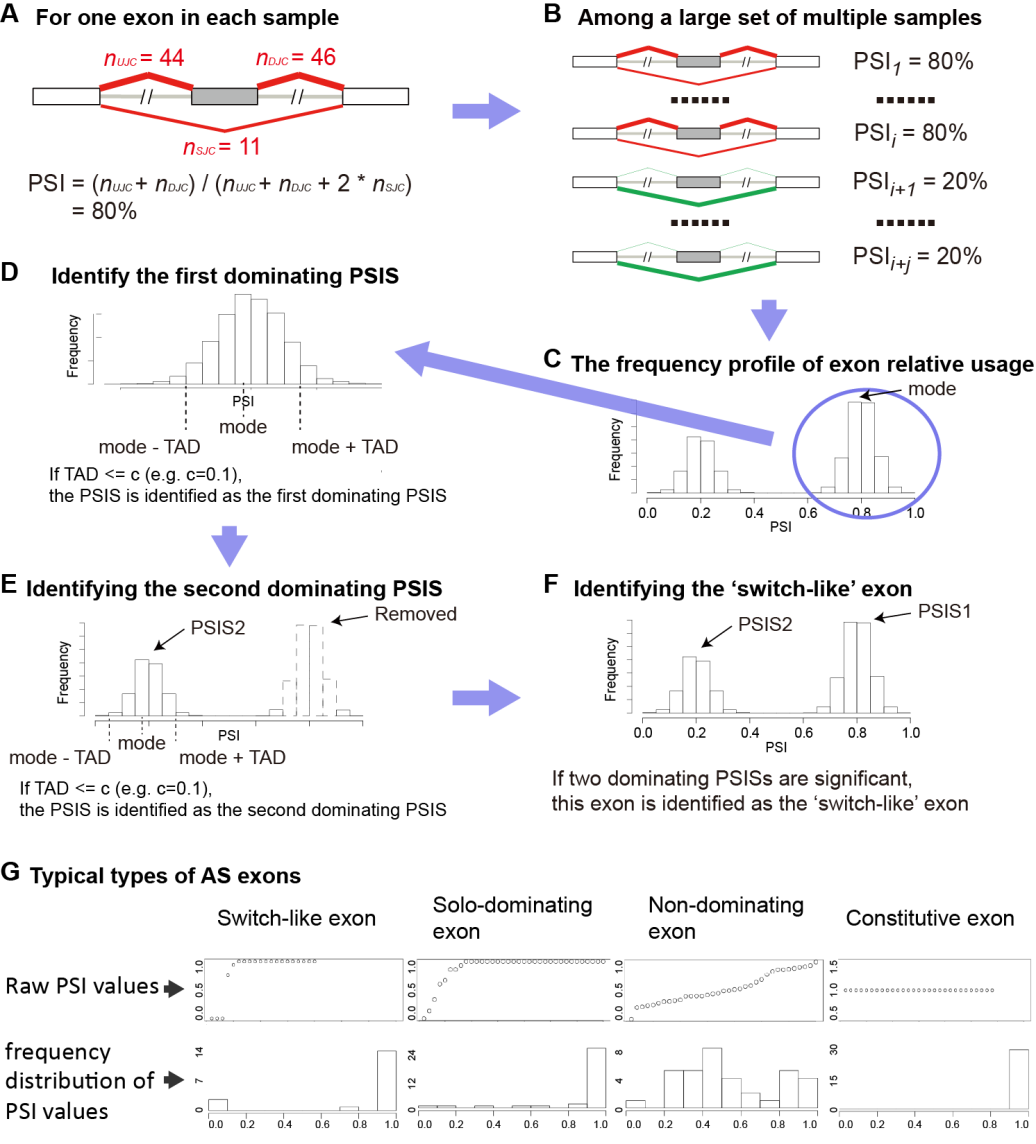
The schematic diagram of iTAD. (A) The calculation of *PSI* as the measurement of exon relative usage. *n*_*UJC*_ and *n*_*DJC*_ are the counts of reads at the exon’s upstream splice junction and its downstream splice junction, respectively. *n*_*SJC*_ is the count of junction reads connecting its upstream and downstream exons but skipping this exon. (B) Calculating PSIs on multiple samples. (C) The histogram of the exon’s relative usage in multiple samples. The mode is identified as the center of a PSIS. (D) The Tertile Absolute Deviation around the mode (TAD) is calculated to describe the intensity of the PSIS. If there are sufficient samples within a narrow region around the mode, reflected by a threshold on the TAD, we identify the PSIS as a dominating PSIS. (E) The identification of the second PSIS after removing samples belonging to ±c region of the first PSIS. (F) The detection of the switch-like exon based on the two PSISs. (G) Four typical relative usage patterns of alternative splicing exons across multiple samples.

The proposed method iTAD is a framework based on a robust statistics to identify switch-like alternative exons as well as exons of other typical usage patterns across multiple samples using RNA-Seq data. The exon relative usage is measured by the inclusion level *PSI* (percent-spliced-in) that calculates the percentage of the transcripts of a gene in a sample are from isoforms including the exon (Fig 1A). PSI values range from 0 to 1. A switch-like exon is one that has high inclusion levels in some samples and low inclusion levels in the rest samples. But this concept need to be more precisely defined. Our method tackles this by studying the distribution of exon inclusion levels among multiple samples using a unsupervised approach.

The input to the method is the read count of isoforms including the studied exon and that of isoforms excluding the exon. For each exon, we calculate the PSI from those two counts in each sample as shown Fig 1A and 1B.

For a gene with multiple isoforms, the PSI or ψ is defined as the expression level of isoforms including this exon divided by the total expression level of all isoforms of this same gene, composed of isoforms including this exon and isoforms skipping this exon [4, 15]. Let *μ*_*I*_ and *μ*_*S*_ denote the expression level of isoforms including this exon and isoforms skipping this exon, respectively. The effective length of one exon is the amount of all possible start sites of splice junction reads in the exon. In RNA-Seq study, *μ*_*I*_ of one isoform can be estimated by the count of reads specifically mapped to this isoform divided by effective length of this isoform, denoted as *n*_*I*_ and *l*_*I*_, respectively. We can also estimate *μ*_*S*_ using *n*_*S*_ divided by *l*_*S*_ in the similar way. For the skipped exon event of alternative splicing as an example (Fig 1A), *n*_*I*_ is the sum of count reads of this exon’s upstream splice junction (denoted as *n*_*UJC*_) and its downstream splice junction (denoted as *n*_*DJC*_). *n*_*S*_ is the count of reads skipping this exon and connecting its upstream and downstream exons, denoted as *n*_*SJC*_. So the effective length *l*_*I*_ is twice of *l*_*S*_. The ψ of a exon can be calculated as Eq 1. Based on this equation, we can calculate the ψ for each exon in each sample.

$$\psi =\frac{\mu_{I}}{\mu_{I}+\mu_{S}}=\frac{n_{I}/l_{I}}{n_{I}/l_{I}+n_{S}/l_{S}}=\frac{n_{UJC}+n_{DJC}}{n_{UJC}+n_{DJC}+n_{SJC}\times 2}$$ (1)

Then we summarize the distribution of the PSIs of this exon across all studied samples using a histogram, and study the pattern of the distribution (Fig 1C). If there is a narrow peak with clear boundary in the distribution, it indicates that there are a specific cluster of samples with very similar relative usage of this exon, which implies shared regulation mechanisms on the alternative splicing of this gene among this group of samples.

We define a “PSI state” (PSIS) to indicate a continuous non-zero region around a local peak in the histogram. The center of PSIS is determined by the mode of all PSIs (Fig 1D). To calculate the mode, we equally divide the PSIs into 11 bins (corresponding to PSIs of 0.0, 0.1, 0.2, …, 0.9, 1.0) and count the frequency in each bin. The one with the largest frequency is selected as the mode of the first PSIS. In the unlikely case of equally largest frequencies in two bins, we take the one with the smaller PSI value as the mode of the first PSIS. To describe the intensity of the PSIS on the histogram, we calculate the absolute deviations from the mode of all samples, and take the region that captures samples with the tertile (1/3 quantile) of the absolute deviation around the mode on both sides as the PSIS region (Fig 1D). This value is called the tertile absolute deviation (TAD) of the distribution, i.e.,

TAD=thefirsttertileof{|x−mode(x)|} (2)

for a random variable *x*. It is a robust measure of the variability of a variable and it is insensitive to outliers, similar to the median around deviation (MAD) estimator in [16]. So TAD is suitable for detecting the PSIS across multiple samples as it is insensitive to random sampling noise in RNA-Seq data.

A narrow PSIS indicates a cluster of samples in which the exon has the same or similar relative usage. We set a threshold *c* to measure how wide a PSIS is, and take a PSIS with TAD less than or equal to *c* as a dominating PSIS (Fig 1D).

We remove the samples belonging to the region of the first PSIS from the histogram, and apply the same method on the remaining samples to identify the second PSIS (Fig 1E). During removing, for a dominating first PSIS, we use the region of ±c around the mode as the region of first PSIS. The same criterion is applied on the 2nd PSIS to decide whether it is a dominating PSIS.

When the number of sequencing reads of an exon in one sample is low, the calculation of PSI can be sensitive to noises. So we take an extra step to validate whether a dominating PSIS is significant by a permutation test to count for the effect of randomness in the sequencing procedure.

To identify a significant PSIS, the null hypothesis is that the TAD value is more than the user-given threshold c. And the alternative hypothesis is that the TAD value is no more than the user-given threshold c.

In each sample, we use the binominal distribution to model the read count of the isoform that includes the exon (*n*_*I*_) given the total read count *n* = *n*_*I*_ + *n*_*S*_ and the exon inclusion level p = ψ, as used in [4, 15]:

$$n_{I}|n,\psi ∼Binominal(n=n_{I}+n_{S},p=\psi =\frac{n_{I}/l_{I}}{n_{I}/l_{I}+n_{S}/l_{S}}).$$ (3)

Based on this binominal distribution (Eq 3) and the TAD function (Eq 2), we use the following steps to calculate the p-value for null hypothesis that the TAD value is more than the user-given threshold c:

1. Given a set of observations of read count *n* and PSI, use this binominal distribution to generate a set of *n*_*I*_, calculate the corresponding PSI values and then calculate the TAD value; repeat the generation process for a set of *n*_*I*_ based on the binominal distribution for 1000 times, then calculate these 1000 simulated TAD values;
2. Compare these 1000 simulated TAD values with the user-given c, and calculate the p-value for the null hypothesis by counting how many TAD values is larger than c.

We use these steps directly to calculate the p-value for each exon. We identify a dominating PSIS as a significant PSIS if the p-value is less than 5%.

The number of significant PSISs is a basic measure for the relative usage pattern of the exon across multiple samples. If the exon has two significant PSISs in the histogram, we identify it as a switch-like exon (Fig 1F). Besides the switch-like relative usage pattern, there are other three typical patterns as shown in Fig 1G, and we call them as solo-dominating exons, non-dominating exons and constitutive exons. Solo-dominating exons have two PSISs but only one is significant. Non-dominating exons have two PSISs but none of them are significant. Constitutive exons have only one PSIS and it is significant.

## Results

### Experiments on simulation data

We conducted simulation experiments to assess the performance of the proposed iTAD method. Experiment 1 was designed to check whether our method can detect the mode of PSIS correctly. In experiment 1 each simulation dataset has only one PSIS, which was assumed to follow the binominal distribution. The modes of PSIS were set as 0.2, 0.6, and 0.99 in three different datasets, respectively. The amount of total reads was set to 100. We used read counts generated from a binominal distribution divided by the total read counts to represent the PSI value of the simulated exon. The probability parameter of the binominal distribution is set as the mode of the PSIS. We also introduced noises that do not belong to the PSIS using the uniform distribution. We tested two different levels of noises for each mode of PSIS: One level was that 80% of PSI values were from the PSIS and 20% were from the uniform noise, and the other level was that 50% of PSIs were from the PSIS and 50% were from the noise. In total, we tested 6 different conditions in experiment 1. In each condition, we generated 100 samples and repeated 1000 times.

Table 1 summarizes the settings of experiment 1 and the estimated parameters by iTAD. The detected modes of PSIS are almost the same as the real modes at different levels of noise. The averages of detected TAD are between 0.01 and 0.04 in different conditions of experiment 1. The averages of the p-values are close to 0 in all simulation conditions. Here, we define the accuracy as the percentage of PSISs correctly identified with p-value less than 5% in one simulation condition. In all the 6 simulation conditions, each detected TAD was smaller than our given threshold (c = 0.1) with significance (p-value < 0.05), and all PSISs are identified successfully. This shows that our method can correctly detect the mode and TAD of a PSIS and identify it as a significant PSIS with high accuracy, even with different levels of noises.

**Table 1:** The settings of experiment 1 and the corresponding parameters calculated by iTAD.

Mode of PSIS	Detected values	100 samples, 100 reads	
		20% noise	50% noise
0.2	Detected mode	0.20	0.20
	Detected TAD	0.02	0.03
	P-value	0.00	0.00
	Accuracy	100%	100%
0.6	Detected mode	0.60	0.60
	Detected TAD	0.03	0.04
	P-value	0.00	0.00
	Accuracy	100%	100%
0.99	Detected mode	1.00	1.00
	Detected TAD	0.01	0.01
	P-value	0.00	0.00
	Accuracy	100%	100%

Then we conducted simulation experiment 2 with three simulation conditions to assess the performance of the proposed iTAD method. Each simulation condition has two PSISs, which were assumed to follow two different binominal distributions. The modes of two PSISs were set as (0.01 and 0.99), (0.8 and 0.99) and (0.2 and 0.5) in three simulation conditions, respectively. We set that 60% of PSIs were from the first PSIS, 30% were from the second PSIS, and 10% were from the uniform noise. Other settings are similar to experiment 1.

Table 2 summarizes the settings of the three simulation conditions and the estimated parameters by iTAD. The detected modes of the first and second PSISs are the almost same as the real modes. The averages of detected TAD are between 0.01 and 0.03 in different simulation conditions. The averages of the p-values are close to 0 in each condition for each PSIS. Every p-values of detected TAD values were less than 5% so all PSISs were correctly identified as the significant PSISs. In all three simulation conditions, switch-like exons are successfully identified by the first and second significant PSISs with always 100% accuracy. This shows that the iTAD can correctly identify switch-like exons with high accuracy, even with the noise. We also conducted simulations with other settings and found that iTAD can work equally well (see S1 Text).

**Table 2:** The settings of experiment 2 and the corresponding parameters calculated by iTAD.

100 samples, 100 reads, 60% PSIS1, 30% PSIS2, 10% noise			
Mode of PSIS (PSIS1, PSIS2)	Detected values	Detected PSIS1	Detected PSIS2
0.01, 0.99	Detected mode	0.00	1.00
	Detected TAD	0.01	0.01
	P-value	0.00	0.00
	Accuracy	100%	100%
0.8, 0.99	Detected mode	0.80	1.00
	Detected TAD	0.03	0.01
	P-value	0.00	0.00
	Accuracy	100%	100%
0.2, 0.5	Detected mode	0.20	0.50
	Detected TAD	0.03	0.03
	P-value	0.00	0.00
	Accuracy	100%	100%

### Application on 16 human body tissues

We applied iTAD on RNA-seq data from the Human Body Map (HBM) 2.0 project, which include 16 different human body tissues with two replicates for each tissue. The 16 tissues are adipose, adrenal, brain, breast, colon, heart, kidney, liver, lung, lymph node, ovary, prostate, skeletal muscle, testes, thyroid, and white blood cells. The annotation of exons were derived from GENCODE transcriptome annotation (v3c) which includes 47,553 genes and 421,368 exons. The annotation of alternative splicing exons for hg19 was derived from the UCSC Alt Event track and downloaded from UCSC genome table browser. Among all exons from GENCODE annotation, we only used the 20,107 cassette exons annotated by UCSC Alt Event in the analyses. More details are provided in the S1 Text.

**Fig 2:**
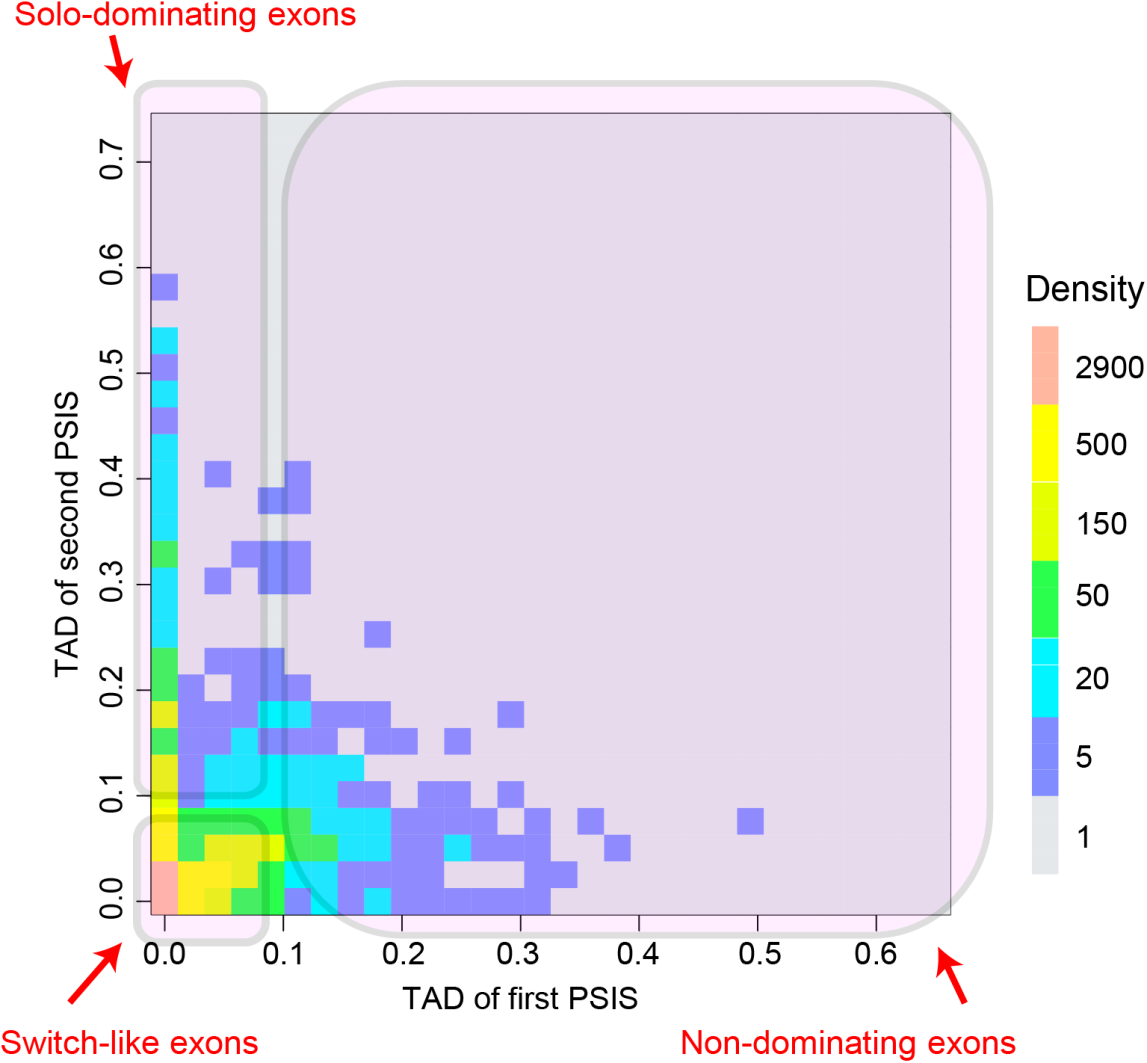
The heatmap of TAD for the first and second PSISs for candidates of exons. The candidates of one exon type represents exons having the corresponding dominating or non-dominating PSISs but before being tested by the significance tests. Switch-like, solo-dominating and non-dominating exons are shown. Constitutive exons are not shown as they only have one PSIS. The “density” on the heatmap shown in colors is the number of exons whose TADs fall into the corresponding region.

We applied iTAD on this dataset to identify the switch-like exon and other 3 types of exons using the default parameters described in the Method. Among all the studied cassette exons, we identified 3,100 (15.4%) switch-like exons, 3,183 (15.8%) solo-dominating exons, 1,336 (6.6%) non-dominating exons and 12,488 (62.1%) constitutive exons. S1 Table provides the full list of identified switch-like exons and their PSIs in the 16 human body tissues. To list the identified switch-like exons ranked by their priorities related with switch-like behaviors, we ranked them by their p-values of the first PSIS from small to large (S1 Table). If the p-values of two exons were equal, we ranked them by the p-value of the second PSIS. In S1 Table for each identified switch-like exon, we provide its chromosome, start site, end site, strand, gene ID, gene name, gene description, mode of each PSIS, significance of each PSIS and PSI value in each sample. There are 1,523 and 1,577 identified switch-like exons on the positive and negative strands, respectively. And there are 2,231 genes containing the identified switch-like exons. The maximum of identified switch-like exons in one gene are 21 exons, which is in the gene TTN at chromosome 2 on the negative strand. Fig 2 shows the heatmap of TAD values for the first and second PSISs for candidate switch-like exons, solo-dominating exons and non-dominating exons. The candidates of one exon type represents exons having the corresponding dominating or non-dominating PSISs but before being tested by the significance tests. Each block represents exons with the corresponding TAD values of the two PSISs, and the color on each block represents the number of exons with the specific TAD values. The three types of exon relative usage patterns can be clearly perceived on the heatmap.

Among the exons on top of the list of detected switch-like exons (S1 Table) with smallest p-values, we found two exons from the same gene DAB1. The first one is the exon 7c of DAB1 studied in [17] (the exon 9c of DAB1 in [18]). It is a 48 bps cassette exon. The modes of the first and second PSISs of this exon is 1 and 0, respectively, and the TAD values of both PSISs are 0 with p-value 0. It is an ideal representative of the switch-like exons identified by iTAD. The significant change of this exon’s relative usage had been observed in early studies on DAB1 from the cortex of mice ranging in age from E10.5 to P10 [17], and during differentiations of P19 cells [19]. The second identified switch-like exon is of 39 bps, and is the exon 7 of DAB1 in [18]. The significant change of this exon’s relative usage had been observed during brain development [18].

### The association of switch-like exons with Alu-intron

We further investigated the relationship between switch-like exons and Alu elements using the detected exon types from the Human Body Map data. Alu elements are one type of transposable elements in primates. It is believed that part of alternative splicing exons are contributed by Alu elements during evolution [20, 21]. We downloaded annotations of Alu elements of hg19 from UCSC genome table browser, and checked their relationship with the detected switch-like exons, solo-dominating exons and non-dominating exons. We calculated the overlapped percentage between each type of exons and Alu elements. For one exon, we selected three specific regions: from upstream 500 bps of its 3’-splice-site (3’ss) to the 3’ss, the exon body region, and its 5’-splice-site (5’ss) to downstream 500 bps. We counted how many exons with a selected region overlapping with Alu elements for each type of exons, and then calculated the Alu present percentage in this type of exons.

**Fig 3:**
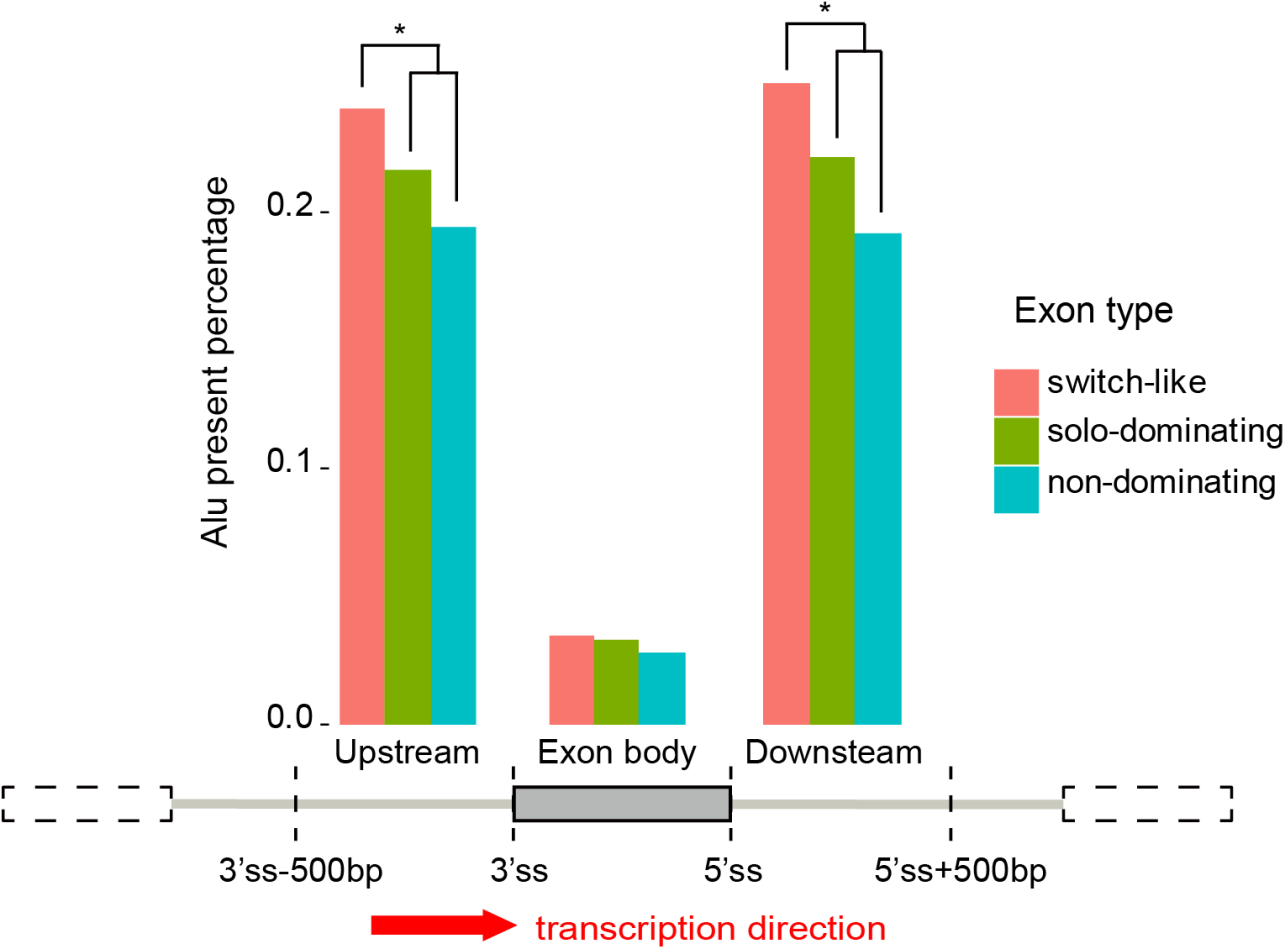
The percentage of Alu elements present in the upstream flanking region, the exon body and the downstream flanking region of each type of exons.

We found that the percentage of switch-like exons with Alu overlap is 3.4%, and the percentages are 3.3% and 2.8% for solo-dominating exons and non-dominating exons, respectively. The slightly more Alu-exons in switch-like exons are not significant (p-value = 0.80 by chi-square test with solo-dominating exons, and p-value = 0.32 by chi-square test with non-dominating exons). On the contrary, associations were observed between switch-like exons and flanking Alu-introns (introns that contain Alu elements). For the upstream 500bp region, the overlapping percentages of Alu elements were 24.0%, 21.6% and 19.4% for switch-like exons, solo-dominating exons and non-dominating exons, respectively. There is a significant increase of Alu-introns in switch-like exons comparing to the other two solo-dominating and non-dominating exons (with chi-square test p-values of 0.08 and 0.01, respectively). For the downstream 500bp region, the overlapping percentages of Alu elements were 25.0%, 22.1% and 19.2% for the three types, respectively, which also shows a significant increase in switch-like exons (with chi-square test p-values of 0.04 and 0.001, respectively). These results are illustrated in Fig 3. These observations indicate that there may not be a significant trend for switch-like exons to be formed by Alu elements, but switch-like exons are more likely to be surrounded with Alu elements in upstream and/or downstream introns.

## Discussion

In this study, we proposed a new method, iTAD, based on a robust statistics for identifying switch-like exons and other types of alternative splicing exons among multiple RNA-seq samples. Unlike existing methods that were designed for detecting the difference of exon relative usages between two groups of samples, iTAD analyzes frequency profiles of exon relative usages among multiple samples in an unsupervised manner to find exons with switch-like usage patterns. Simulation studies showed that iTAD can identify the switch-like exon with high accuracy even when there are noises. Applying iTAD on 16 different human body tissues, we identified 3,100 significant switch-like exons. We observed that there is not a significant trend for switch-like exons to be formed by Alu elements, but switch-like exons are more likely to be associated with Alu elements in their flanking introns. Previous study found that the insertion of Alu elements can change the splicing pattern and lead to the conversion from constitutive exons to alternative splicing exons [22]. Our observation suggests that many intron splicing enhancers or silencers regulating switch-like exons may be provided by Alu elements. The experiments show that iTAD is a useful tool for detecting switch-like exons and other exon relative usage patterns from multiple samples. The software package is available upon email requests.

## Supporting information

S1 Table The full list of identified switch-like exons and their PSIs in human body tissues. (XLS)

S1 Text The supplementary methods and results. (DOCX)
