# Supplementary material for: The identification of switch-like alternative splicing exons among multiple samples with RNA-Seq data
Source: PLoS One. 2017 May 25;12(5):e0178320. doi: 10.1371/journal.pone.0178320 (PMC5444801; doi:10.1371/journal.pone.0178320)
Supplement: S1 Text — (DOCX) [file pone.0178320.s002.docx]

The identification of switch-like alternative splicing exons among multiple samples with RNA-Seq data

Zhiyi Qin^1^, Xuegong Zhang^1,2,*^

^1^: MOE Key Laboratory of Bioinformatics, Bioinformatics Division and Center for Synthetic and Systems Biology, TNLIST / Department of Automation, Tsinghua University, Beijing, China

^2^: School of Life Sciences, Tsinghua University, Beijing, China

**Supplementary Methods**

**Processing of RNA-seq Dataset**

The RNA-Seq dataset we used in this study is collected from the Illumina Human Body Map 2.0 project. The raw reads were downloaded from the NCBI GEO database (GSE30611, http://www.ncbi.nlm.nih.gov/geo/query/acc.cgi?acc=GSE30611). There are 32 samples of 16 human tissues and 3 samples of mixed tissues. We didn’t include those 3 samples of mixed tissues in this study. Each body tissue has two replicates. Their libraries were extracted from the whole cell fraction. One of them was sequenced with 75-bps single-end reads, and the other one was sequenced with 50-bps paired-end reads. Each sample contained about 70 million reads on average. We treated the paired-end reads as two single-end reads in each sample in our study.

The Grape pipeline (downloaded from http://big.crg.cat/services/grape) [[1](#_ENREF_1)] was used to map the raw reads to the reference genome and to control the quality of the data. It implemented the GEM software [[2](#_ENREF_2)] for the mapping step. The UCSC hg19 was used as the reference genome. The default parameters of the Grape pipeline were used.

The annotation of exons were derived from GENCODE transcriptome annotation (v3c) [[3](#_ENREF_3)]. An exon will be filtered if there is no reads aligned to this exon among every tissues. The annotation of alternative splicing exons for hg19 was derived from the UCSC Alt Event track and downloaded from UCSC genome table browser (http://genome.ucsc.edu/cgi-bin/hgTables). We only used the type of cassette exons annotated by UCSC Alt Event track in the following analysis.

**Supplementary Results**

*Simulation S1: Simulation of one PSIS*

Firstly, we conducted a simulation S1 that there is only one PSIS among multiple samples. We simulated the condition, in which there is a gene with a cassette exon containing two isoforms. We assumed that given total reads, the reads count of included isoforms in this simulated gene follows a binominal distribution. So we can use the reads count generated by a binominal distribution divided by total reads count to calculate the PSI value of this AS exon. The probability of the binominal distribution is set as the mode of PSIS. We test different modes of PSIS including 0.01, 0.2, 0.4, 0.6, 0.8 and 0.99.

We also test the influence of sample size and the total reads counts. For total reads counts, we simulated two different conditions: 10 (few reads) and 100 (much reads). We simulated two different sample size: 10 samples (small size of samples) and 100 samples (large size of samples). In each condition, we repeat 1000 times to show whether the result is robust and calculate their accuracy. Here accuracy is the percentage of the PSIS correctly identified with p-value less than 5% among all cases. We use the default threshold c with 0.1 and p-value cutoff with 5% in following simulations.

We showed the average of detected modes of PSIS among 1000 times of repeats, the average of detected TAD values, the average of p-values in each condition and the accuracy.

**Table 1. The results of simulation S1.**

| **Mode of PSIS** | **Detected values** | **10 samples** | | **100 samples** | |
| --- | --- | --- | --- | --- | --- |
|  |  | **10 reads** | **100 reads** | **10 reads** | **100 reads** |
| 0.01 | Detected mode | 0.00 | 0.00 | 0.00 | 0.00 |
|  | Detected TAD | 0.00 | 0.00 | 0.00 | 0.00 |
|  | P-value | 0.00 | 0.00 | 0.00 | 0.00 |
|  | Accuracy | 100% | 100% | 100% | 100% |
| 0.2 | Detected mode | 0.17 | 0.20 | 0.17 | 0.20 |
|  | Detected TAD | 0.03 | 0.02 | 0.07 | 0.02 |
|  | P-value | 0.04 | 0.00 | 0.00 | 0.00 |
|  | Accuracy | 74.1% | 100% | 100% | 100% |
| 0.4 | Detected mode | 0.37 | 0.40 | 0.39 | 0.40 |
|  | Detected TAD | 0.05 | 0.02 | 0.10 | 0.02 |
|  | P-value | 0.13 | 0.00 | 0.09 | 0.00 |
|  | Accuracy | 17.1% | 100% | 32.2% | 100% |
| 0.6 | Detected mode | 0.58 | 0.60 | 0.61 | 0.60 |
|  | Detected TAD | 0.05 | 0.02 | 0.10 | 0.02 |
|  | P-value | 0.15 | 0.00 | 0.07 | 0.00 |
|  | Accuracy | 14.1% | 100% | 42.0% | 100% |
| 0.8 | Detected mode | 0.80 | 0.80 | 0.83 | 0.80 |
|  | Detected TAD | 0.03 | 0.02 | 0.07 | 0.02 |
|  | P-value | 0.05 | 0.00 | 0.00 | 0.00 |
|  | Accuracy | 68.1% | 100% | 100% | 100% |
| 0.99 | Detected mode | 1.00 | 1.00 | 1.00 | 1.00 |
|  | Detected TAD | 0.00 | 0.00 | 0.00 | 0.00 |
|  | P-value | 0.00 | 0.00 | 0.00 | 0.00 |
|  | Accuracy | 100% | 100% | 100% | 100% |

We simulated the condition, in which there is a gene with a cassette exon containing two isoforms. We assumed that given total reads, the reads count of included isoforms in this simulated gene follows a binominal distribution. The probability of the binominal distribution is set as the mode of PSIS. The total reads count is the amount of reads mapped to included isoforms and excluded isoforms. We test different modes of PSIS including 0.01, 0.2, 0.4, 0.6, 0.8 and 0.99. We simulated two different sample size: 10 samples (small size of samples) and 100 samples (large size of samples). For total reads counts, we simulated two different conditions: 10 (few reads) and 100 (much reads). It is showed the average of detected modes of PSIS, the average of detected TAD values, the average of p-values in each condition and the accuracy. Accuracy is the percentage of cases correctly identified among all cases.

In simulation S1, no matter which the condition is applied, the detected mode can be almost the same as the real mode of PSIS (Table 1). In most conditions of simulation S1, the detected TAD is no larger than the threshold (c = 0.1). Here, both of read counts and the sample size can affect the detected TAD obviously. The null hypothesis is to test whether the TAD is larger than user-given threshold c. In the conditions of 100 reads, the results is always significant. While with 10 reads, the result is not always significant. Here the p-value also is affected by both of reads counts and the sample size. But reads counts affect the p-value much more than the sample size. The accuracy for conditions with 100 reads is always 100%. So with high-level of reads counts, the TAD and the significance calculation method can be used to identify the significant PSIS with high accuracy.

**References**

1. Knowles DG, Roder M, Merkel A, Guigo R (2013) Grape RNA-Seq analysis pipeline environment. Bioinformatics 29: 614-621.

2. Marco-Sola S, Sammeth M, Guigo R, Ribeca P (2012) The GEM mapper: fast, accurate and versatile alignment by filtration. Nat Methods 9: 1185-1188.

3. Consortium EP (2012) An integrated encyclopedia of DNA elements in the human genome. Nature 489: 57-74.
